# Mathematical Modelling to Assess the Impact of Lockdown on COVID-19 Transmission in India: Model Development and Validation

**DOI:** 10.2196/19368

**Published:** 2020-05-07

**Authors:** Bakiya Ambikapathy, Kamalanand Krishnamurthy

**Affiliations:** 1 Department of Instrumentation Engineering Madras Institute of Technology Campus Anna University Chennai, Tamil Nadu India

**Keywords:** covid-19, coronavirus, epidemic, mathematical modelling, pandemic, sars

## Abstract

**Background:**

The World Health Organization has declared the novel coronavirus disease (COVID-19) to be a public health emergency; at present, India is facing a major threat of community spread. We developed a mathematical model for investigating and predicting the effects of lockdown on future COVID-19 cases with a specific focus on India.

**Objective:**

The objective of this work was to develop and validate a mathematical model and to assess the impact of various lockdown scenarios on COVID-19 transmission in India.

**Methods:**

A model consisting of a framework of ordinary differential equations was developed by incorporating the actual reported cases in 14 countries. After validation, the model was applied to predict COVID-19 transmission in India for different intervention scenarios in terms of lockdown for 4, 14, 21, 42, and 60 days. We also assessed the situations of enhanced exposure due to aggregation of individuals in transit stations and shopping malls before the lockdown.

**Results:**

The developed model is efficient in predicting the number of COVID-19 cases compared to the actual reported cases in 14 countries. For India, the model predicted marked reductions in cases for the intervention periods of 14 and 21 days of lockdown and significant reduction for 42 days of lockdown. Such intervention exceeding 42 days does not result in measurable improvement. Finally, for the scenario of “panic shopping” or situations where there is a sudden increase in the factors leading to higher exposure to infection, the model predicted an exponential transmission, resulting in failure of the considered intervention strategy.

**Conclusions:**

Implementation of a strict lockdown for a period of at least 21 days is expected to reduce the transmission of COVID-19. However, a further extension of up to 42 days is required to significantly reduce the transmission of COVID-19 in India. Any relaxation in the lockdown may lead to exponential transmission, resulting in a heavy burden on the health care system in the country.

## Introduction

The novel coronavirus disease (COVID-19) pandemic spread through 190 countries within 20 weeks from the epicenter of Wuhan in China, affecting 334,000 populations and causing more than 14,500 deaths by mid-March 2020. As of April 6, 2020, the number of infected people has increased to 1,210,956 and the number of associated deaths to 67,594 [1]. While several European countries had gone through stage 3 of the pandemic by the second week of February, India entered into transition towards stage 3 at this point in time. It is well known that there are differences in behavior regarding the epidemic within the same country and in other countries; mathematical modelling helps to predict the course of the epidemic to determine why there is no uniformity in the infection [2,3]. In addition, the health system can utilize the predictions of such models as an intelligent tool to decide on the types of control measures as well as the time and location of their application. It is also essential to understand the dynamics of the transmission of an infection introduced in a new country or location and to forecast whether the proposed control measures will result in measurable effects [4,5]. The measured effects suggest that alternate interventions should be designed; therefore, it is obvious that predictions must be critically analyzed before applying interventions [6].

India recorded its first case of COVID-19 infection on January 30, 2020, in a student from China’s epicenter, Wuhan [7]. The Ministry of Health in India initiated the course of action of screening travelers in airports and then shut down schools during the first week of March 2020. As of March 22, 2020, India reported only 360 positive COVID-19 cases from 23 states across the country [8]. However, compared to the course of the epidemic in western countries, either the epidemic in India progressed through a slow phase or the number of asymptomatic cases in India is higher. The Government of India imposed the Janata Curfew for 24 hours as an initial measure to contain the spread of infection, followed by a lockdown under the Disaster Management Act 2005 for a period of 21 days starting on March 24, 2020 [9]. In the absence of an effective vaccine for COVID-19 prevention, the only remaining options are prevention of further influx of migrant cases at airports and seaports and contact tracing. China learned from its experience that only complete shutdown prevented further spread, and Italy learned from its experience that negligence of communities towards simple public health strategies leads to uncontrolled morbidity and mortality.

It is well known that COVID-19 infection leads to mild and self-limiting respiratory symptoms. However, two betacoronaviruses, severe acute respiratory syndrome coronavirus (SARS-CoV) and Middle East respiratory syndrome coronavirus (MERS-CoV), result in severe forms of pneumonia, causing 10% and 37% mortality, respectively [10,11]. SARS-CoV spread through 26 countries and affected more than 8000 individuals, while the MERS-CoV epidemic was mainly focused in Middle Eastern countries and affected nearly 2500 people.

In recent studies, it has been reported that the maximum time from the onset of coronavirus infection to hospitalization is 10 days, with an incubation period of 2 to 14 days [12,13]. According to the World Health Organization (WHO), the time between the start of symptomatic manifestations and death is approximately 2-8 weeks [14]. Another study reports that the duration of viral shedding is 8-37 days [15]. Further, the effectiveness of the interventions depends on multiple factors, and a recent report recommends estimating the optimal periods to implement each intervention [16]. However, most countries have implemented 14 days of self-quarantine to prevent further spread of the infection. Therefore, it is important to mathematically estimate the lockdown period required to interrupt the transmission of COVID-19 infection with respect to each country because the contact patterns between individuals are highly dynamic and nonhomogeneous across each population. SARS-CoV can survive on inanimate objects such as metal, wood, paper, glass, and cloth for 4-5 days at room temperature [17]. It has been shown that clinically ill patients play a vital role in SARS-CoV transmission [18], as peak viral load in the respiratory tract occurs approximately ten days after the onset of symptoms [19].

Recently, the Government of India took the very intelligent step of implementing a lockdown for a period of 21 days starting at midnight on March 14, 2020. Based on this scenario, we developed a mathematical model to predict the course of the epidemic in India and to determine the impact of the intervention under different possible conditions.

## Methods

**Figure 1:**
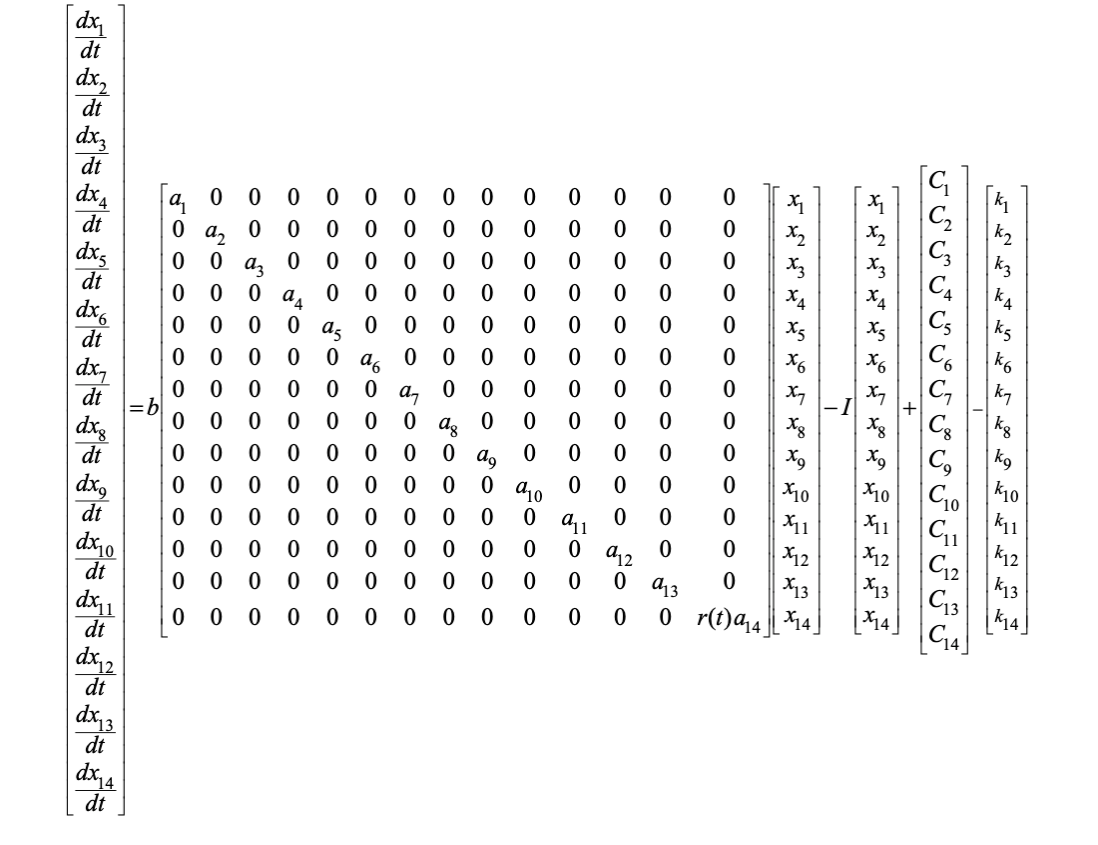
Framework of the first order ordinary differential equations used in the model.

In this work, a dynamic mathematical model for prediction of the future infected population with COVID-19 was developed. Infected populations in the date range from February 19, 2020 to March 18, 2020 served as inputs for the development of the model. The WHO Situation Reports on COVID19 and updates by the International Society for Infectious Diseases (ISID) were the major sources of the numbers of cases in different countries [20,21]. The infected populations from 14 countries (China, Italy, Germany, France, the United States, the United Kingdom, Sweden, the Netherlands, Austria, Canada, Australia, Malaysia, Singapore, and India) were considered for the development of the model due to the major interactions and travel of infected populations between these countries and India for education and employment. The first case reported in India was related to medical education in Wuhan, China; also, countries such as Italy and Germany admitted groups of students in February 2020. The developed model consists of a framework of first order ordinary differential equations of the form shown in Figure 1.

The model has the constraint 0 ≤ *x_i_(t)* ≤ *TP_i_*, where *x_i_(t)*, *i* = 1, 2, ..., 14 is the total number of infected people at time *t* for each country. **
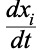
**, *i* = 1, 2, ..., 14 is the rate of change of the infected population at time *t* for each country. *a_i_*, *i* = 1, 2, ..., 14 is the parameter that influences the rate of infection in each country. *C_i_*, *i* = 1, 2, ..., 14 is the parameter of the model that is influenced by factors specific to each country, such as population density and cross-antibodies. *b* is a parameter common to all the considered countries. *TP_i_*, *i* = 1, 2, 14 is the total population in each country, and *r*(*t*) is a random change acting on the infection dynamics due to sociological factors. *I* is the identity matrix. Finally, *k_i_*(*t*), *i* = 1, 2, ..., 14 is the forcing function that represents the intervention in terms of travel restrictions such as lockdown, medications, and vaccination strategies. However, at present, because travel restrictions are the existing intervention strategy, it was considered appropriate to apply “lockdown” as the intervention. Furthermore, the total population of each country was considered as the maximum susceptible population for COVID-19 infection. We considered the whole population for the prediction because the rate of RNA positivity is only 1/307 (0.3%) in blood samples, indicating very minimal viremia; the antibody positivity in the community is not known at this point in time, and variable results would be obtained for R_0_. The parameters of the model were estimated using the numbers of reported infected cases provided by the WHO, which are available as open source data. A prediction error method [22] was utilized for the estimation of the model parameters using the reported cases. The developed model was validated using the reported infections in the adopted period and was utilized to predict the future infected cases in the 14 considered countries up to a further period of 65 days.

After the validation, the developed model was utilized to determine the impact of the intervention strategy in terms of lockdown for India to contain the infection. Five different intervention strategies with travel lockdown periods of 4 days, 14 days, 21 days, 42 days, and 60 days (Multimedia Appendix 1) were analyzed using the developed model. Further, a random increase in exposure to infection on the day before the implementation of the intervention strategy due to aggregation of the susceptible population in locations such as grocery stores, markets, railway stations, and buses due to panic shopping, etc., was considered, and these scenarios were analyzed. Three different scenarios with increases in infection exposure by factors of 2, 3, and 5 were considered on the day before the start of the lockdown (Multimedia Appendix 2).

## Results

The developed model can capture the infection dynamics in each country to a considerable extent and predict future cases (Multimedia Appendix 3 and 4). Also, the correlation between the reported cases and those obtained using the developed model was found to be high for all 14 countries (China: 0.9763, Italy: 0.9960, Germany: 0.9416, France: 0.9965, USA: 0.9992, UK: 0.9959, Sweden: 0.9615, Netherlands: 0.9976, Austria: 0.9979, Canada: 0.9987, Australia: 0.9971, Malaysia: 0.8769, Singapore: 0.9751, India: 0.9858).

**Figure 2:**
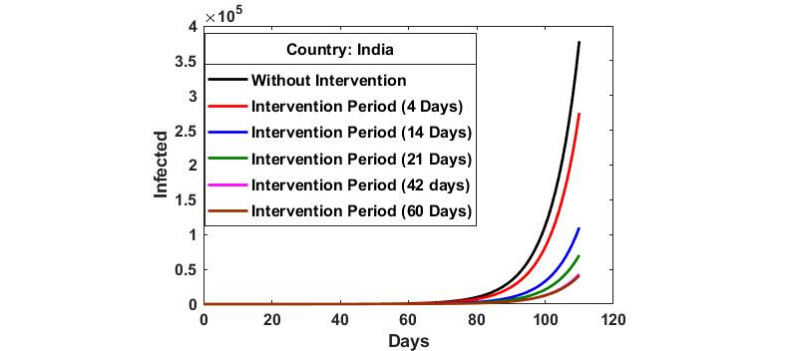
The effects of the intervention periods on the number of infected cases in India according to the model.

The infected populations predicted using the developed model for the case of India along with the effects of the intervention periods of 4, 14, 21, 42, and 60 days are presented in Figure 2. No significant change was observed in the predicted infected cases with a 4-day intervention period compared to the scenario without intervention. However, there were significant decreases in the number of infected cases with intervention periods of 21, 42, and 60 days. For the 21-day lockdown intervention, the number of predicted cases was reduced from 378,036 (non-intervention) to 70,424 at 110 days. For a 42-day lockdown intervention, the predicted cases were further reduced significantly to 42,950. However, there was no significant change in the predicted number of infections between the 42-day and 60-day intervention scenarios.

**Figure 3:**
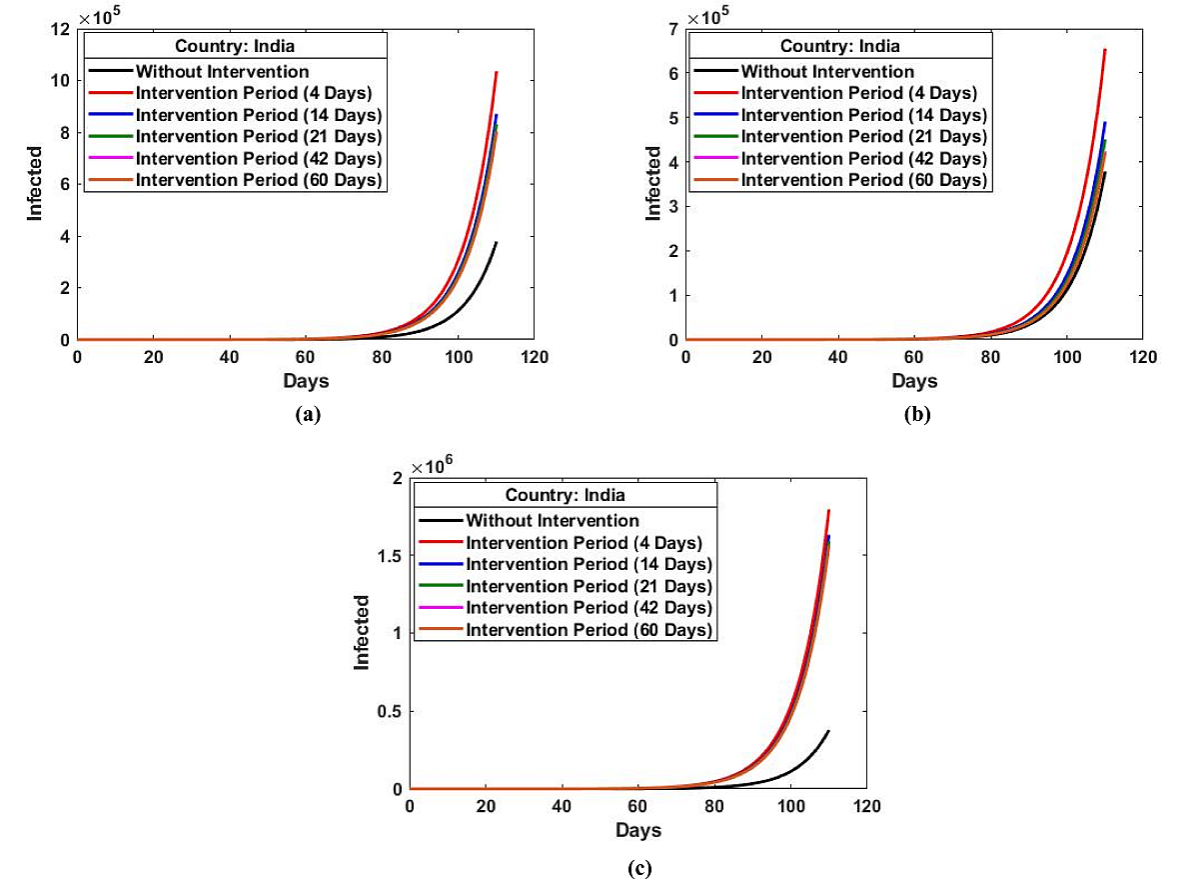
The effects of the random changes in the infection dynamics due to social activity on the day before the intervention period for (a) r=2, (b) r=3, and (c) r=5.

Figure 3 (a-c) shows the effects of the random changes in the infection dynamics on the day before the intervention period with *r*=2, 3, and 5, respectively, on the number of predicted infections. It was observed that even for 2-fold augmentation in transmission (*r*=2), the predicted number of infected people increased exponentially to 450,618 despite the 21-day intervention. The predicted number of infected people further increased exponentially for the 3-fold (*r*=3) and 5-fold (*r*=5) augmentations in transmission.

## Discussion

### Principal Findings

The results obtained using the developed model suggest that the implementation of a 21-day lockdown is necessary to slow the progression of the COVID-19 epidemic in India. An increase in the lockdown period up to 42 days is required to significantly reduce the number of COVID-19 cases in the country. However, the impact of the intervention depends on the extent to which the exposure to COVID-19 was augmented due to aggregations of the susceptible population with the infected population just before the lockdown. Even 2-fold augmentation may result in exponential transmission in different parts of the country.

Although the COVID-19 pandemic has spread to 190 countries worldwide, other than China, 10 European countries have been the most affected [23]. Currently, the epidemic is heading towards stage 3 in the second most populated country in the world, India, and the course of the epidemic in India is expected to determine the global burden of morbidity and mortality as well as the future course of this pandemic. In the present model, we included the cases reported for a period of 45 days from 13 countries that are employment and education hubs for Indians, considering the fact that many exposed people must have migrated to India and escaped screening. However, the epidemic in India did not set in until March 2020, as seen from the number of reported cases [20]. This can be explained by the experiences in the Chinese epidemic, particularly at the epicenter, Wuhan, where it was found that a single case cannot trigger an epidemic and that the introduction of several cases is required to generate a successful epidemic [24]. India started to report additional cases only in the second week of March 2020. This provided an opportunity to construct a model to predict future cases; we preferred to restrict the prediction to a shorter period of 110 days, assuming that other factors will alter the epidemic behavior and affect the long-term prediction capability of the model.

Several models are used to analyze the dynamics of an epidemic. Of these, the Susceptible-Infected-Recovered, Susceptible-Exposed-Infected-Recovered, and Susceptible-Infected-Susceptible models are frequently explored by epidemiologists and public health experts [25-27]. Most of the available epidemiological models assume the network of interactions [28]. Furthermore, there are several challenges and limitations when these models are applied to a new infection of pandemic proportions [29]. To date, the possibility of reinfection due to severe acute respiratory syndrome coronavirus 2 (SARS-CoV-2) has not yet been analyzed, and superinfections cannot be described by the Susceptible-Infected-Recovered model. Also, most of the available models are time-invariant [30]. Furthermore, when a very large population is considered, the Susceptible-Infected-Recovered model suffers from numerical errors because the number of susceptible people in the initial days of the epidemic is very high and the numbers of infected and recovered people in the first few days are low.

In the present work, we attempted to predict the progression of the COVID19 epidemic in the first few months of the outbreak. In view of the exponential transmission and almost uniform spread in several countries, we considered a simple mathematical framework consisting of ordinary differential equations due to their higher dynamic prediction capabilities and the ease of applying control forces to the model to find the outcomes. Furthermore, such models have been proved to be highly useful in epidemiological and population modelling for effective prediction of future populations.

Based on the evidence of viral survival on surfaces, incubation period, viral shedding duration by infected persons, and the datewise reported cases from the 14 studied countries, we considered lockdowns for 4, 14, 21, 42, and 60 days as intervention strategies. In addition, we assumed that an intervention approach of adopting only lockdown would have a 30% impact on transmission. With these inputs, our model predicted that with a 21-day lockdown, there will be a significant break in transmission; it also predicted that this can be even further improved with a 42-day lockdown. Further extension to 60 days may not result in a desirable impact on transmission. Therefore, the lockdown imposed by the Government of India is likely to have a significant impact on containing the COVID-19 epidemic in the country. Using the IndiaSim model, Eili Klein et al [31] predicted that sensitivity of the virus to temperature and humidity will result in decreased transmission in India. In IndiaSim, the authors assumed that a 21-day lockdown would have a 25% impact on transmission. In our model, we assumed a 30% reduction of transmission; in addition, the infection rate was considered to influence the transmission at least 10 times more than other parameters, such as temperature and humidity.

Although India has started to implement interventions in the form of travel restrictions, on the day before the implementation of the intervention, an unusual increase in social gatherings was witnessed throughout the country; this must have changed the epidemic dynamics to a great extent. It has been established that the viral droplet nuclei can travel up to 2 meters and that the virus can remain infective in the atmosphere for several hours [32]. In our model, we assumed that even a small number of people newly infected with COVID-19 in such a population would alter the transmission 2-fold, 3-fold, or even 5-fold. For these situations, the model predicts exponential transmission, as seen in other European countries. In real situations, this will be revealed if the testing strategy is extended to screen the asymptomatic population in communities.

### Limitations

The first limitation of this study is that it considers the total population in each country as the susceptible population because sufficient evidence of the fraction of each population that is susceptible to SARS-CoV-2 is not yet available. The model was constructed by including the reported cases based on the initial testing strategy adopted in India with the assumption that there was no community spread until the first week of March 2020. Also, the model utilized in this study has limited dynamic prediction range; therefore, the authors restricted the prediction to a window of 110 days. The model will need to be updated with the numbers of reported cases to analyze the future course of the infection after May 21, 2020.

### Conclusion

Our model suggests that strict implementation of a country-wide 21-day lockdown in India will reduce community transmission, and an extension of another 21 days (total period of 42 days) will further improve the break in the transmission chain in local communities. This will also provide an opportunity to identify the proportion of SARS and case fatality rates. Health facilities in India can be reorganized to handle SARS and to reduce the case fatality rate. Therefore, the government must impose a lockdown with stringent measures, preferably for at least 42 days. Despite these stringent measures, approximately 40,000 cases will be spread throughout the country. Contact tracing and community screening must be completed during this period, and the lockdown must be lifted in a phased manner at the district level. Any flexibility in implementing the lockdown or sudden release from lockdown and failing to achieve contact tracing may lead to exponential transmission, leading to large numbers of COVID-19 cases that India will not be able to handle with the available health infrastructure and professional staff.

The results derived from the developed model for lockdown intervention strategies in India can provide useful insight into the imposition and release of lockdown to slow the progression of the COVID-19 epidemic in other countries which are currently in stage 1 or stage 2 of the epidemic. As of April 24, 2020, 23077/38522 (59.9%) of the confirmed COVID-19 infections in the WHO-defined South-East Asia region are in India [33], and Indonesia and Bangladesh may enter stage 3 of the epidemic, similar to India, in another 2-4 weeks. These countries must impose intervention strategies well before stage 3 and continue proper testing strategies and contact tracing to contain the epidemic effectively.

## Appendix

Multimedia Appendix 1 Supplementary Figure S1.

Multimedia Appendix 2 Supplementary Figure S2.

Multimedia Appendix 3 Supplementary Figure S3.

Multimedia Appendix 4 Supplementary Figure S4.

## Abbreviations

COVID-19: coronavirus disease
ICMR: Indian Council of Medical Research
ISID: International Society for Infectious Diseases
MERS-CoV: Middle East respiratory syndrome coronavirus
SARS-CoV: severe acute respiratory syndrome coronavirus
SARS-CoV-2: severe acute respiratory syndrome coronavirus 2
VCRC: Vector Control Research Centre
WHO: World Health Organization
