# Supplementary figures and images for: Mathematical Modelling to Assess the Impact of Lockdown on COVID-19 Transmission in India: Model Development and Validation
Source: JMIR Public Health Surveill. 2020 May 7;6(2):e19368. doi: 10.2196/19368 (PMC7207014; doi:10.2196/19368)

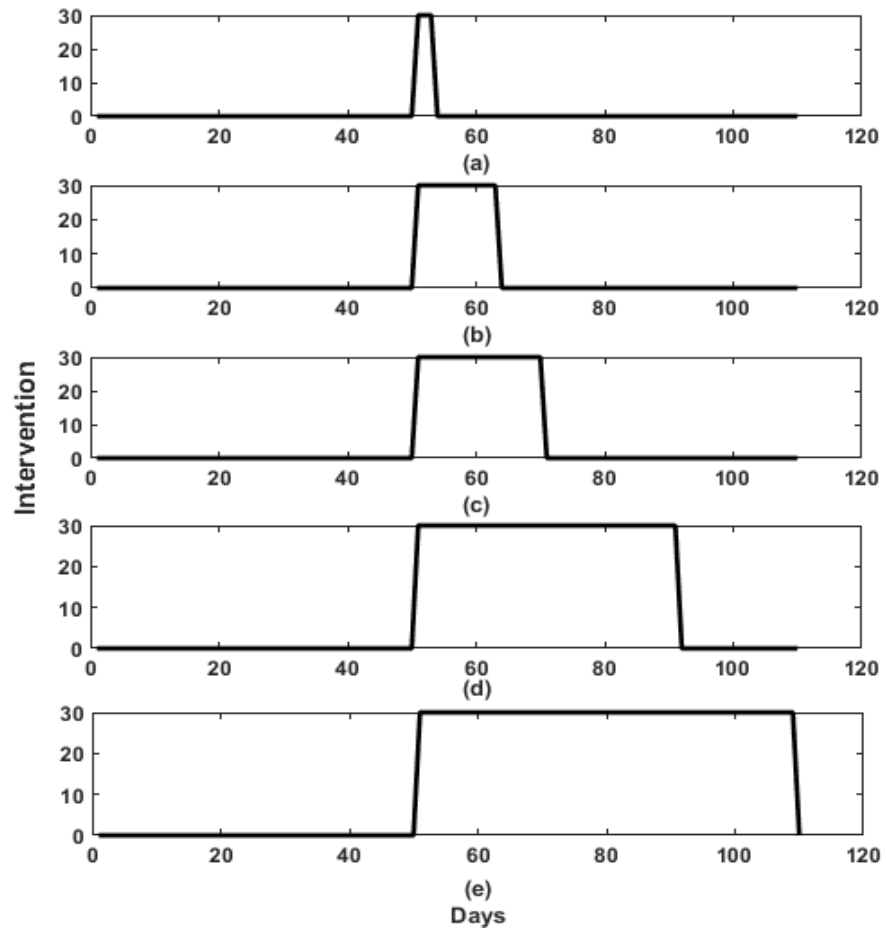

**Figure S1. The considered intervention periods of (a) 4 days, (b) 14 days, (c) 21 days, (d) 42 days and (e) 60 days**

Supplement: Multimedia Appendix 1 [file publichealth_v6i2e19368_app1.pdf]
