# Supplementary material for: Mathematical Modelling to Assess the Impact of Lockdown on COVID-19 Transmission in India: Model Development and Validation
Source: JMIR Public Health Surveill. 2020 May 7;6(2):e19368. doi: 10.2196/19368 (PMC7207014; doi:10.2196/19368)
Supplement: Multimedia Appendix 2 [file publichealth_v6i2e19368_app2.pdf]

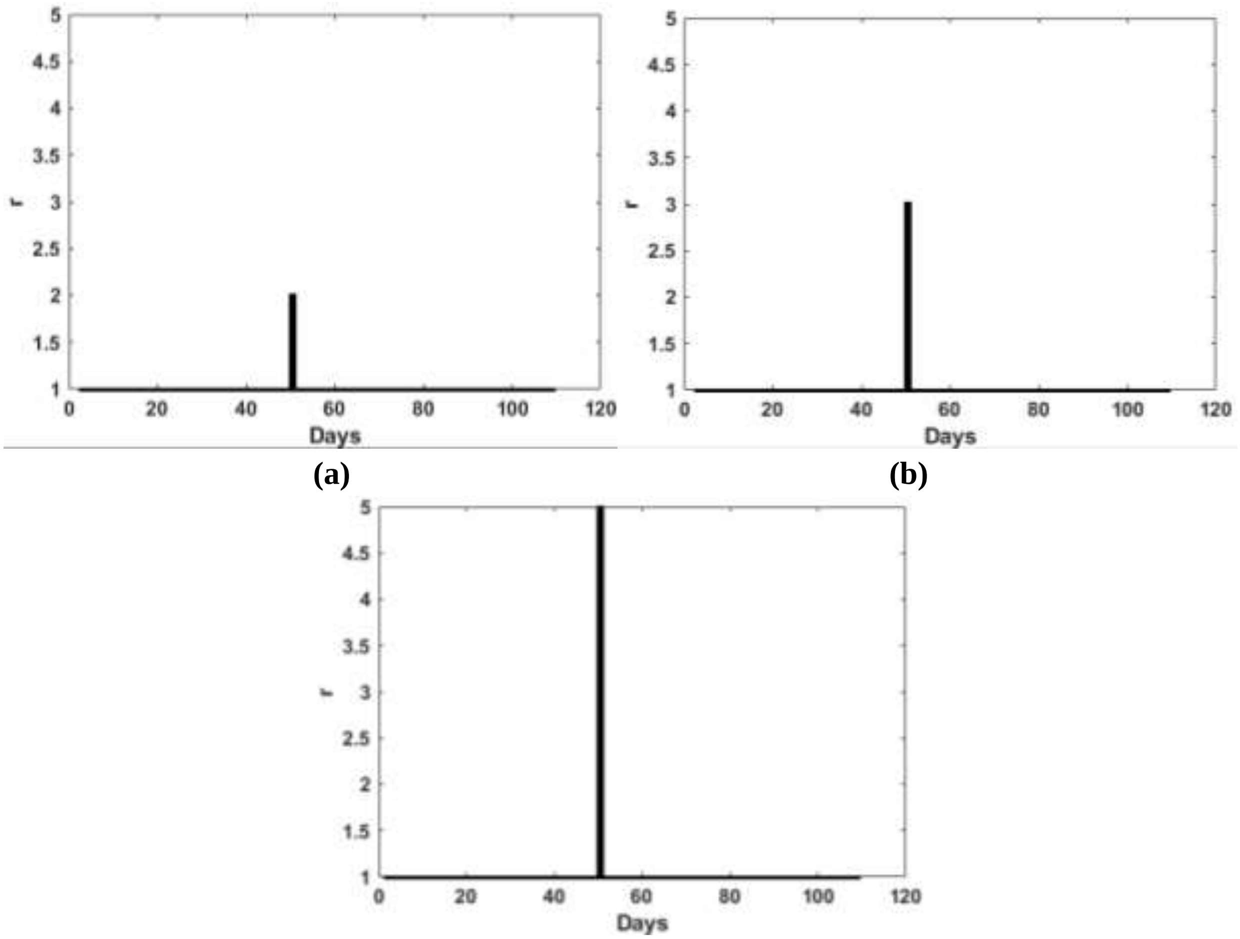

(a) (b) (c)

Figure S2. Three different scenarios with increase in infection dynamics by factors of (a) 2, (b) 3 and (c) 5 on the day before the start of travel intervention
