# Supplementary material for: Mathematical Modelling to Assess the Impact of Lockdown on COVID-19 Transmission in India: Model Development and Validation
Source: JMIR Public Health Surveill. 2020 May 7;6(2):e19368. doi: 10.2196/19368 (PMC7207014; doi:10.2196/19368)
Supplement: Multimedia Appendix 3 [file publichealth_v6i2e19368_app3.pdf]

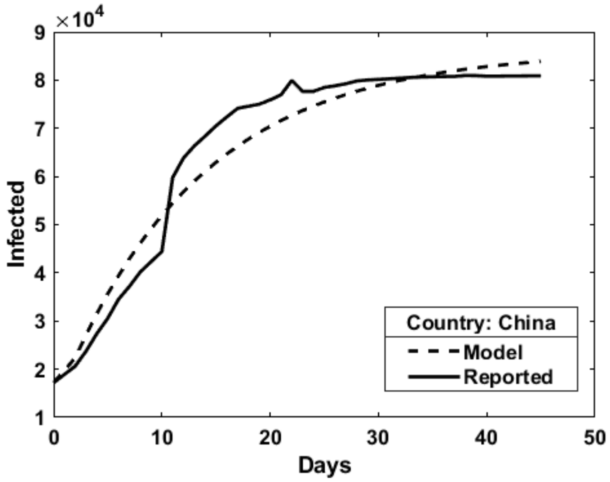
×10^4
Infected
Days
Country: China
Model
Reported

(a)

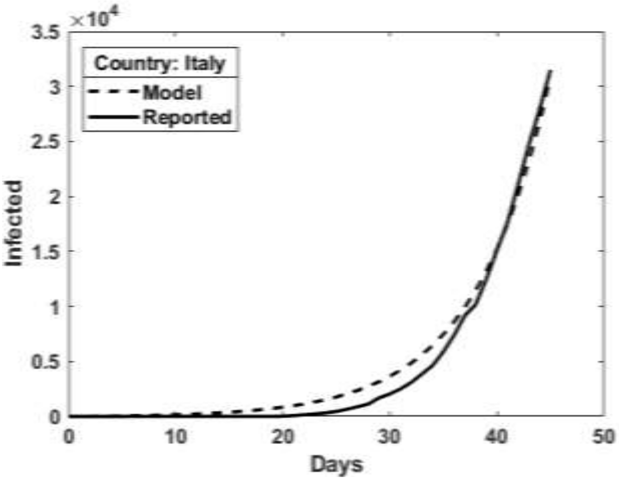
×10^4
Infected
Days
Country: Italy
Model
Reported

(b)

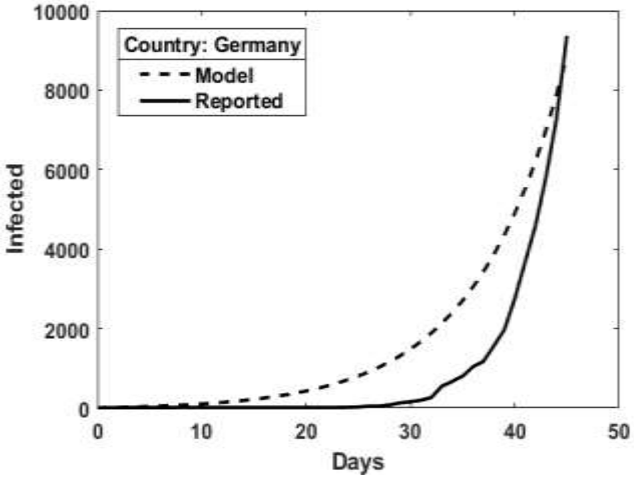
Infected
Days
Country: Germany
Model
Reported

(c)

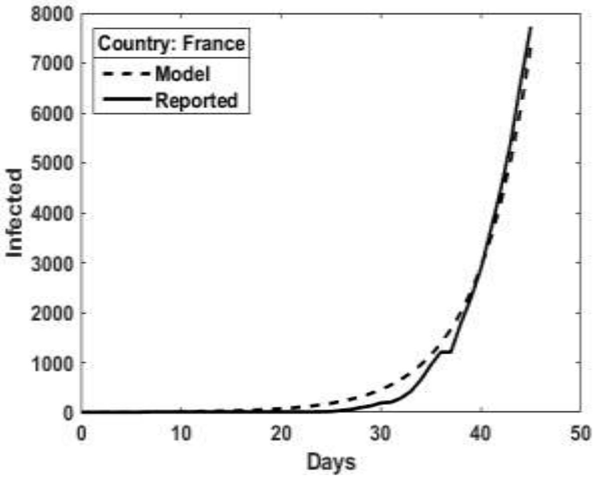
Infected
Days
Country: France
Model
Reported

(d)

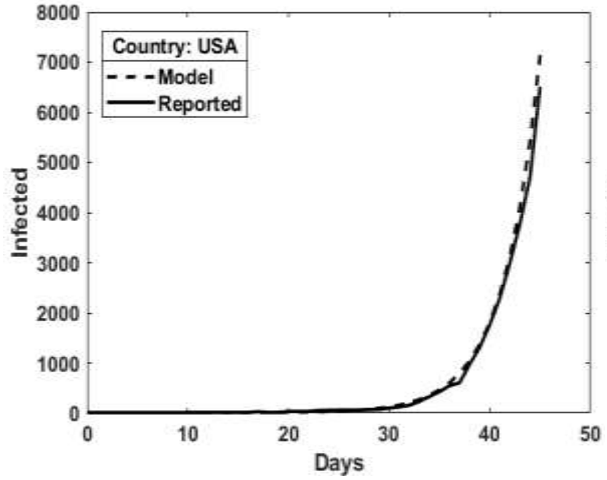
Infected
Days
Country: USA
Model
Reported

(e)

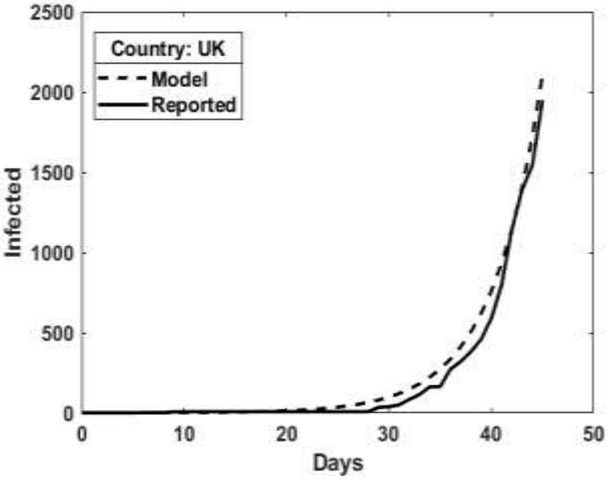
Infected
Days
Country: UK
Model
Reported

(f)

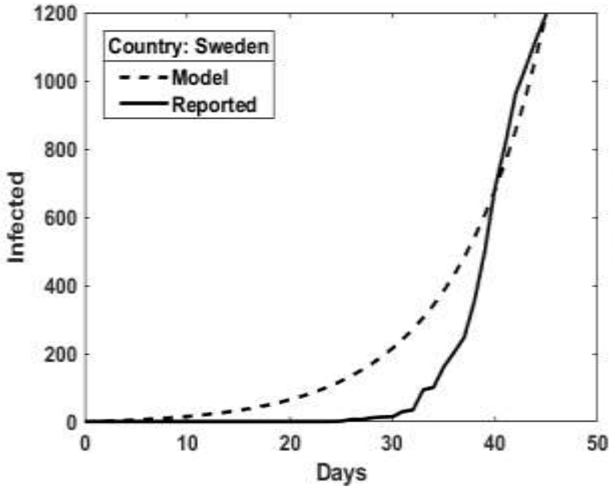
Country: Sweden
Model
Reported
Infected
Days
0
200
400
600
800
1000
1200
0
10
20
30
40
50

(g)

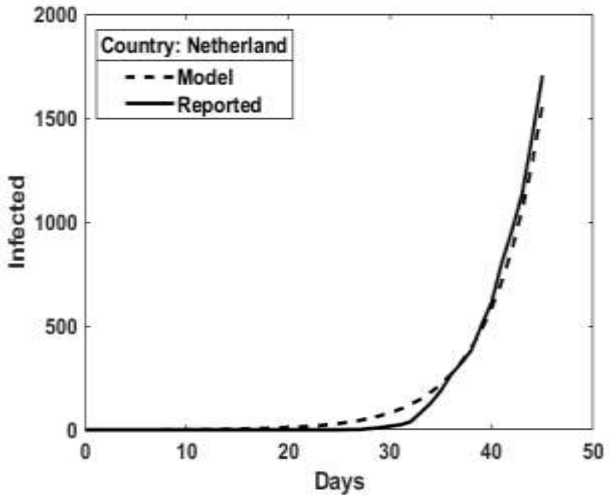
Country: Netherland
Model
Reported
Infected
Days
0
500
1000
1500
2000
0
10
20
30
40
50

(h)

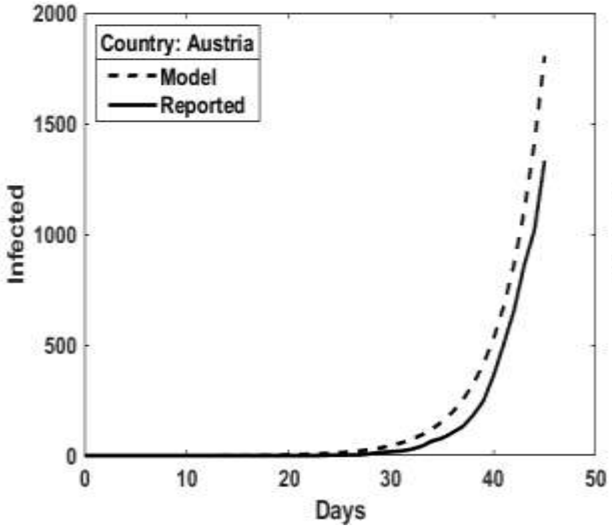
Country: Austria
Model
Reported
Infected
Days
0
500
1000
1500
2000
0
10
20
30
40
50

(i)

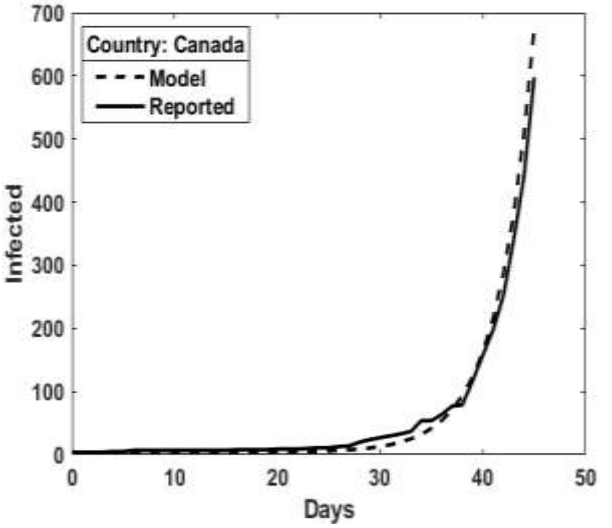
Country: Canada
Model
Reported
Infected
Days
0
100
200
300
400
500
600
700
0
10
20
30
40
50

(j)

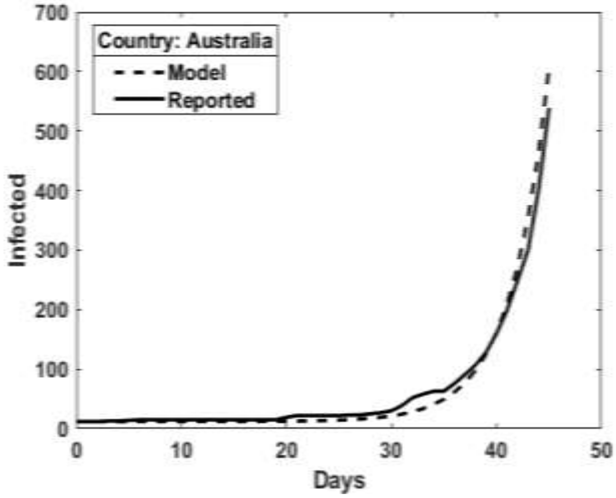
Country: Australia
Model
Reported
Infected
Days
0
100
200
300
400
500
600
700
0
10
20
30
40
50

(k)

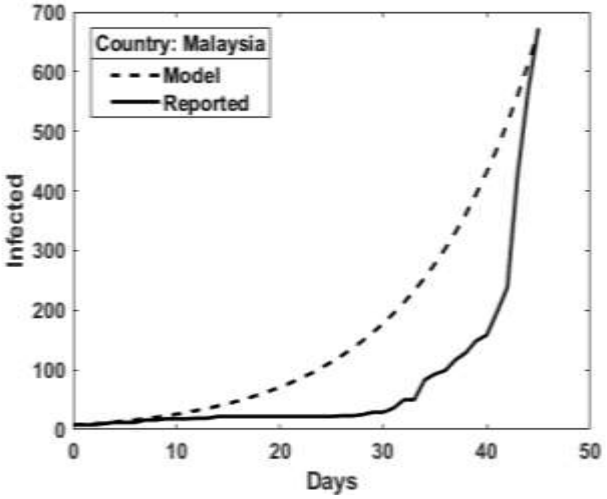
Country: Malaysia
Model
Reported
Infected
Days
0
100
200
300
400
500
600
700
0
10
20
30
40
50

(l)

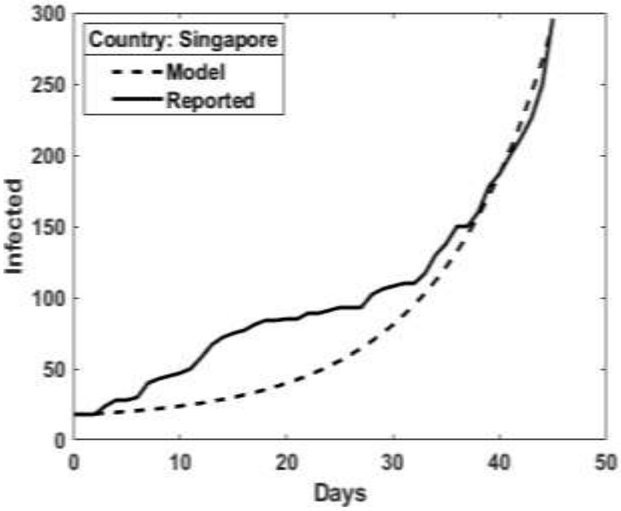

(m)

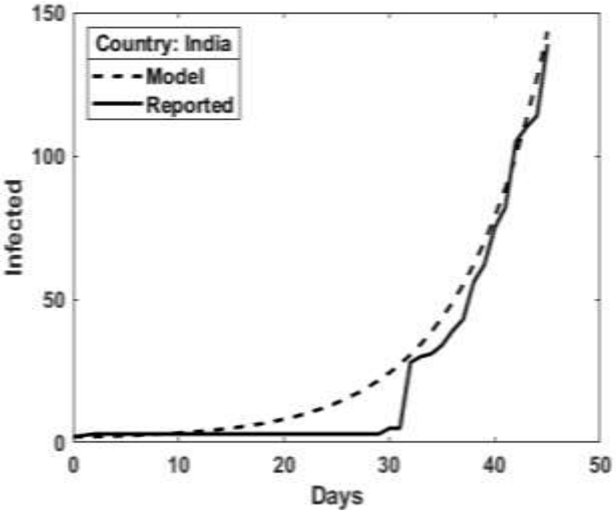

(n)

Figure S3. The Infected population predicted using the developed model and the actual reported cases in (a) China, (b) Italy, (c) Germany, (d) France, (e) USA, (f) UK, (g) Sweden, (h) Netherland, (i) Austria, (j) Canada, (k) Australia, (l) Malaysia, (m) Singapore and (n) India (Day 0: 2nd February 2020)
