# Supplementary material for: Mathematical Modelling to Assess the Impact of Lockdown on COVID-19 Transmission in India: Model Development and Validation
Source: JMIR Public Health Surveill. 2020 May 7;6(2):e19368. doi: 10.2196/19368 (PMC7207014; doi:10.2196/19368)
Supplement: Multimedia Appendix 4 [file publichealth_v6i2e19368_app4.pdf]

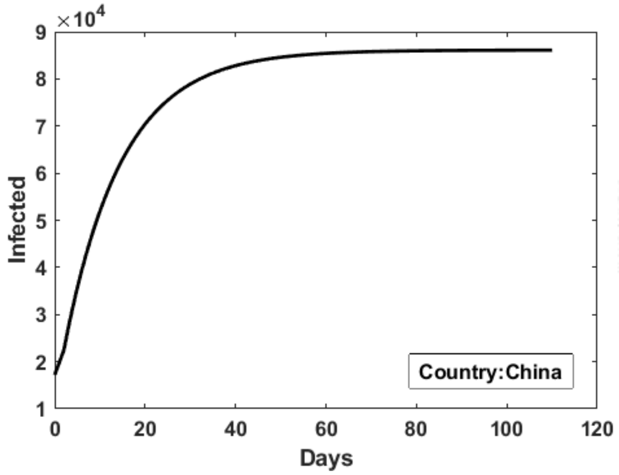
$\times 10^4$
Infected
Days
Country:China
9
8
7
6
5
4
3
2
1
0
20
40
60
80
100
120

(a)

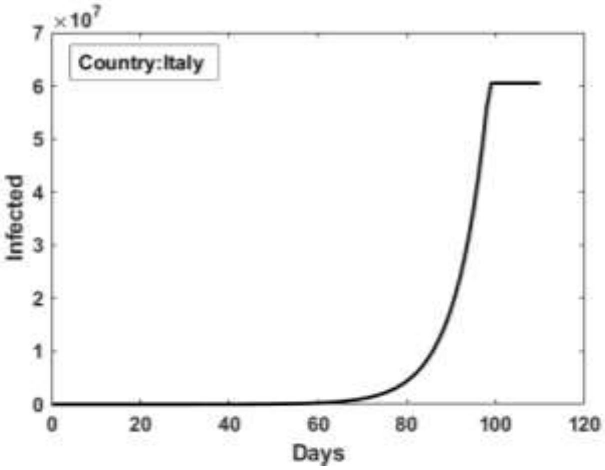
$\times 10^7$
Infected
Days
Country:Italy
7
6
5
4
3
2
1
0
0
20
40
60
80
100
120

(b)

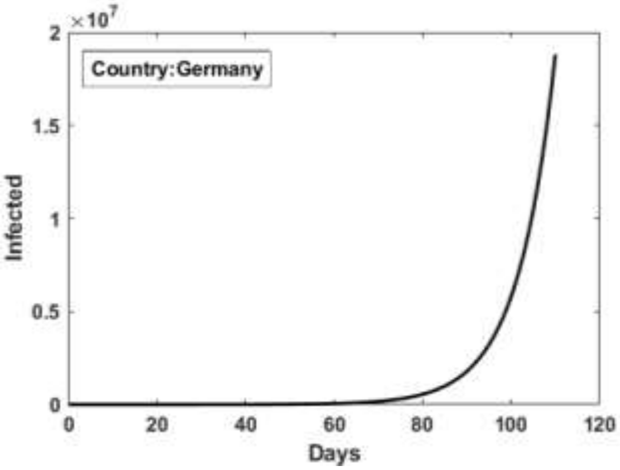
$\times 10^7$
Infected
Days
Country:Germany
2
1.5
1
0.5
0
0
20
40
60
80
100
120

(c)

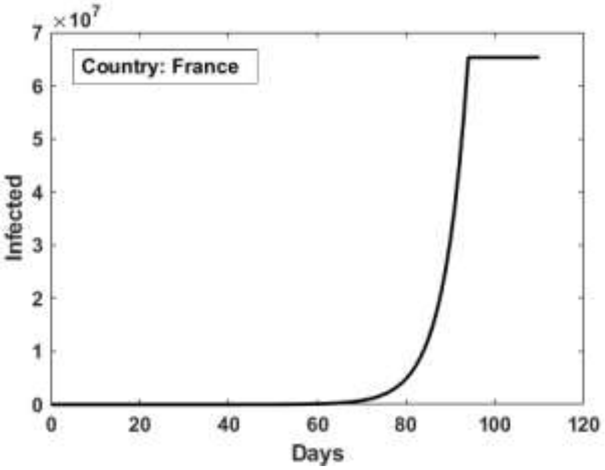
$\times 10^7$
Infected
Days
Country: France
7
6
5
4
3
2
1
0
0
20
40
60
80
100
120

(d)

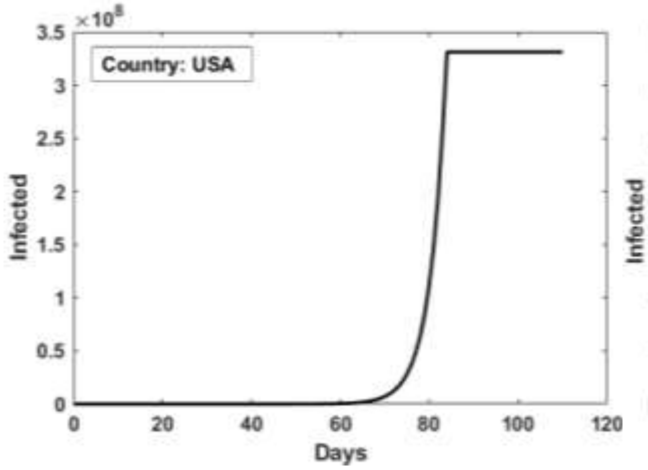
$\times 10^8$
Infected
Days
Country: USA
3.5
3
2.5
2
1.5
1
0.5
0
0
20
40
60
80
100
120

(e)

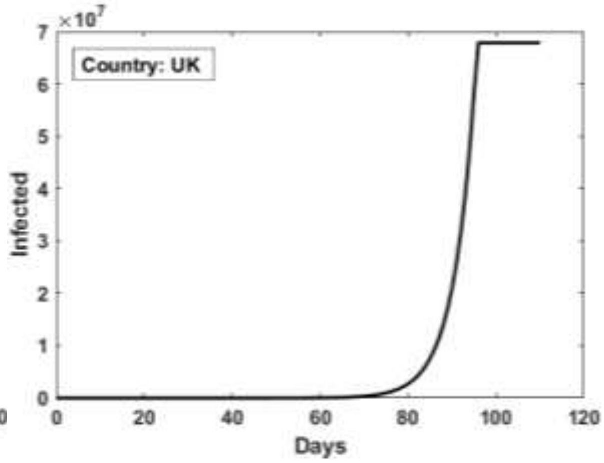
$\times 10^7$
Infected
Days
Country: UK
7
6
5
4
3
2
1
0
0
20
40
60
80
100
120

(f)

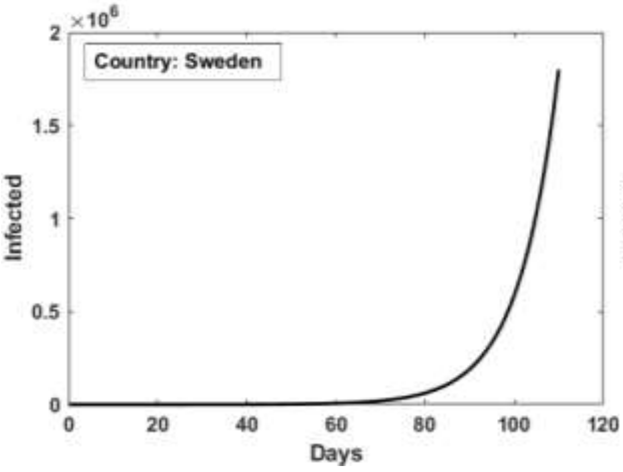
×10^6
Country: Sweden
Infected
Days

(g)

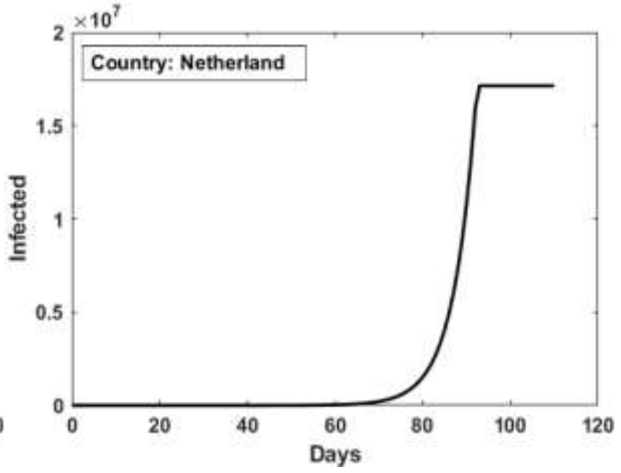
×10^7
Country: Netherland
Infected
Days

(h)

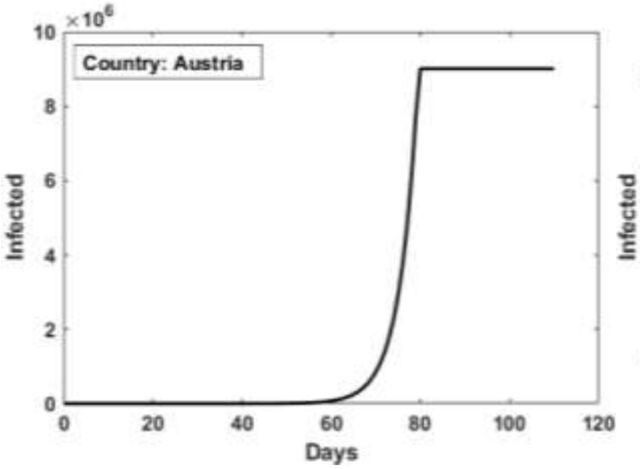
×10^6
Country: Austria
Infected
Days

(i)

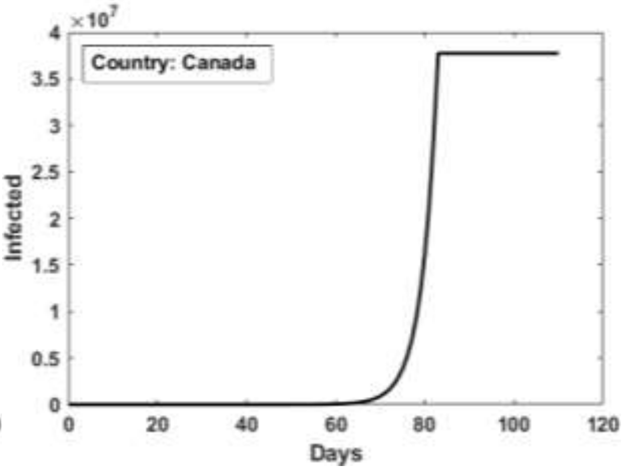
×10^7
Country: Canada
Infected
Days

(j)

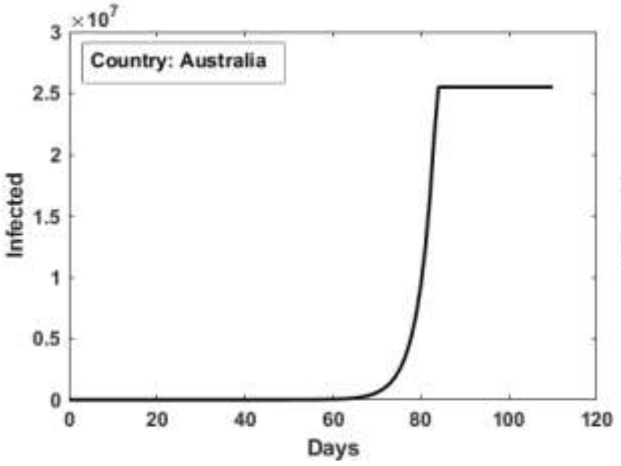
×10^7
Country: Australia
Infected
Days

(k)

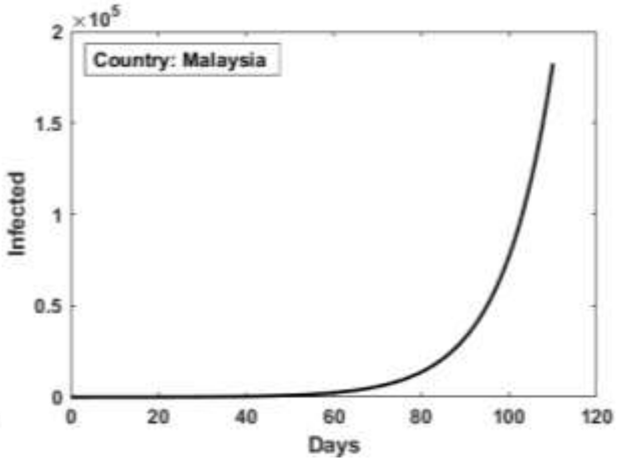
×10^5
Country: Malaysia
Infected
Days

(l)

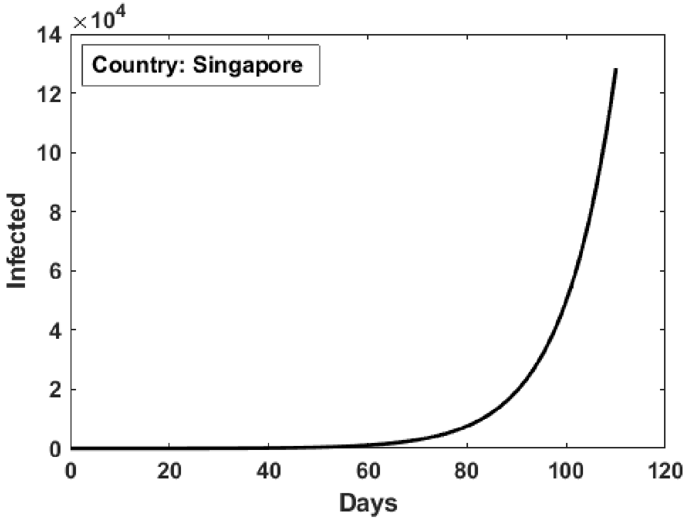

(m)

Figure S4. The Infected population predicted using the developed model for (a) China, (b) Italy, (c) Germany, (d) France, (e) USA, (f) UK, (g) Sweden, (h) Netherland, (i) Austria, (j) Canada, (k) Australia, (l) Malaysia and (m) Singapore, without intervention. (Day 0: 2nd February 2020)
